# Sources of awareness, perceptions, and use of JUUL e-cigarettes among adult cigarette smokers

**DOI:** 10.1371/journal.pone.0238377

**Published:** 2020-09-01

**Authors:** Julia Cen Chen-Sankey, Andy S. L. Tan, Meghan Bridgid Moran, Samir Soneji, Stella J. Lee, Kelvin Choi

**Affiliations:** 1 Division of Intramural Research, National Institute on Minority Health and Health Disparities, Bethesda, MD, United States of America; 2 Annenberg School for Communication, University of Pennsylvania, Philadelphia, PA, United States of America; 3 Department of Health, Behavior and Society, Johns Hopkins University Bloomberg School of Public Health, Baltimore, MD, United States of America; 4 Department of Health Behavior, Gillings School of Global Public Health, University of North Carolina, Chapel Hill, NC, United States of America; 5 Department of Media and Communication, Konkuk University, Seoul, Republic of Korea; University of Calfornia San Francisco, UNITED STATES

## Abstract

**Introduction:**

Given JUUL e-cigarettes’ potential for smoking cessation and its drastically increased sales in the U.S., more evidence is needed to understand the antecedents of JUUL use among adult cigarette smokers. This study assessed the relationships between awareness sources, perceptions about using JUUL, and JUUL use behavior.

**Methods:**

In an online study with adult smokers who were aware of JUUL e-cigarettes (n = 341), respondents reported their sources for learning about JUUL, perceptions of using JUUL versus Vuse (a competitor brand), and ever and past-30-day (current) JUUL use. Multivariable logistic regressions were used to examine the associations between awareness sources, perceptions, and JUUL use, adjusting for covariates.

**Results:**

Learning about JUUL through internet ads was associated with positive perceptions about JUUL compared to Vuse, including JUUL was more fun to use (AOR = 2.04, 95% CI = 1.21, 3.42) and tastier (AOR = 1.96, 95% CI = 1.19, 3.22). Perceiving JUUL as being tastier (AOR = 2.07, 95% CI = 1.23, 3.49), more helpful for quitting smoking (AOR = 2.07, 95% CI = 1.22, 3.53), and cooler (AOR = 2.07, 95% CI = 1.21, 3.56) than Vuse was associated with ever using JUUL. Only perceiving JUUL as being tastier (AOR = 1.98, 95% CI = 1.10, 3.59) than Vuse was associated with current use of JUUL.

**Discussion:**

Adult smokers may be more likely to focus on the sensory and social experience of using JUUL rather than JUUL’s smoking cessation benefits. These positive perceptions are likely to be influenced by internet ads in general instead of JUUL’s official marketing outlets. They are also more likely to sustain JUUL use than JUUL’s perceived smoking cessation benefits.

## Introduction

Five years since its launch, JUUL, a small, rechargeable e-cigarette device manufactured by JUUL Labs, has become the leading e-cigarette brand in the U.S. Unlike traditional e-cigarette products that use free-base nicotine, JUUL products use nicotine salt derived from loose-leaf tobacco, which enables high efficiency in nicotine delivery and makes inhaling higher levels of nicotine less harsh for the user [1]. Due to this design, JUUL may produce a more satisfying user experience even at high nicotine concentrations than traditional e-cigarettes and combustible cigarettes [2]. With increased popularity in the U.S. market, the sales of JUUL increased from US$2.2 to US$16.2 million between 2016 and 2017 alone [3]. As of July 2018, JUUL represented more than 70% of the U.S. convenience store market for e-cigarette products and is currently considered the e-cigarette “brand leader” [4]. A U.S. national representative survey showed that in 2018, about 10% of youth (ages 15–17) and 11% of young adults (ages 18–21) reported ever using JUUL, and 6% and 8%, respectively, reported past-30-day use [5]. The same study showed that JUUL use is more prevalent among combustible tobacco users than those who do not use combustible tobacco. Among smokers aged 15–34, 14% and 10% had ever used JUUL and used JUUL in the past 30 days, respectively [5]. Another recent study found that in 2018, among adult combustible tobacco users aged 18–54 in the U.S., 15% and 12% had ever used JUUL and used JUUL in the past 30 days, respectively [6].

JUUL Labs has claimed that the product is an “alternative to smoking” [7]. A few studies funded by JUUL Labs have suggested that using JUUL e-cigarettes may indeed serve as an effective smoking cessation method for adult smokers given its high efficiency of nicotine delivery and flavors [8,9]. Other research funded by the U.S. National Institute on Drug Abuse and the U.S. Food and Drug Administration suggested that JUUL use alone may be less harmful than smoking cigarettes or using other types of e-cigarettes, producing lower levels of toxicants including free radicals and carbonyls [10]. Therefore, JUUL may be a less harmful alternative to cigarette smoking if further research demonstrates its effectiveness in promoting smoking cessation as well as reduced harmfulness and addictiveness compared to cigarettes.

One potential influencing factor that shapes the perceptions and behavior of using JUUL among adult smokers is the sources of awareness about JUUL products. Exposure to e-cigarette marketing—one major source of learning about e-cigarettes—has been found to be one of the important predictors for e-cigarette use [11–15]. Investigating sources of awareness may help us understand how JUUL garnered such popularity in a short period of time; investigating the association between use of these sources and JUUL-use behavior provides valuable information on potential communication and policy strategies for regulating JUUL e-cigarettes and its marketing practices. One potential mechanism by which e-cigarette marketing and sources of awareness influence product use is promoting favorable perceptions towards e-cigarettes [16–18]. Evidence has shown that JUUL Labs’ advertising and promotional practices have included the placement of materials on myriad media platforms including websites, social media sites, magazines, radio, and TV [19–21]. Most of these advertisements have depicted attractive lifestyles and attitudes (e.g., relaxation, freedom) and conveyed sex appeal, while simultaneously making claims about the potential efficacy of the product for smoking reduction and cessation [19–21]. Other sources of awareness including word-of-mouth from friends and family as well as news from TV and radio may also influence perceptions and behavior of using tobacco products [22].

Since 2018, health advocacy groups have urged JUUL Labs to halt its marketing practices that attract teenagers [23] and the FDA has warned the JUUL Labs to stop its outreach to youth [24]. Evidence has shown that JUUL’s particular marketing practices have attracted tobacco-naïve teenagers to experiment with the product [21], which may further lead to nicotine addiction and progression to cigarette smoking [25]. Since then, JUUL has terminated its advertising through its social media accounts and using young models [26]. However, little is known whether the influence of these marketing practices on adult smokers’ perceptions and behavior of using JUUL products. Given JUUL’s potential benefit in reducing harm among adult cigarette smokers, it is critical to understand whether certain sources of awareness are more influential in shaping adult smokers’ perceptions about JUUL and whether these perceptions are associated with their JUUL use behavior. This type of evidence will be useful to serve as the guidelines for regulatory agencies to consider its e-cigarette marketing policies and communication messages as they relate to encourage adult cigarette smokers to completely transition to use e-cigarette products.

Using an online sample of current adult cigarette smokers (≥18 years old) who were aware of JUUL e-cigarettes, this study aimed to assess [1] the associations between receiving JUUL-related information through various channels (including marketing outlets) and perceptions about JUUL e-cigarettes; and [2] the relationships between JUUL-specific positive perceptions and JUUL use behavior. Findings from this analysis will clarify JUUL-related perceptions and use, and inform the development of tobacco marketing regulations that minimize public health risks and maximize public health benefits related to e-cigarette use.

## Methods

### Study sample

An online panel of adult current cigarette smokers (≥18 years old) was collected through Survey Sampling International (SSI) in April 2018. The study eligibility criteria were: residing in the U.S., having smoked ≥100 cigarettes in their lifetime, and currently smoking some days or every day in the past 30 days. Targeted email invitations were sent by SSI to potentially eligible panelists based on their socio-demographic information and cigarette smoking status. Respondents consented online and completed a survey about tobacco beliefs and use intentions. The survey took about 20 minutes to complete. Of 1,182 panelists who started the survey, 913 met the eligibility criteria (77.2%), and among those, 803 (87.9%) completed the survey. Of the 803 adults, 341 (42.5%) answered “yes” when asked if they had “heard of JUUL before today”; only these respondents were included in this analysis. Respondents who completed the survey were rewarded points by SSI that can be exchanged for cash. The study was approved by the Harvard University T.H. Chan School of Public Health Institutional Review Board (Protocol number 18–0467).

### Measures

#### Sources of awareness about JUUL

Among those who were aware of JUUL e-cigarettes, respondents were asked to report at least one source of awareness about JUUL e-cigarettes through the question “How did you learn about JUUL?” Ten options describing specific sources were used and were grouped into five categories: (1) General advertising (print media ads, internet ads including online banners and social media, direct mail/email); (2) JUUL brand advertising (JUUL sponsored events, JUUL outdoor ads, JUUL website or social media account); (3) Social circle (word-of-mouth from friends and family, friends and family’s social media accounts); (4) News stories on TV/radio/online, and (5) Physical stores.

#### JUUL-related perceptions

Respondents were asked to compare perceptions of JUUL e-cigarettes against those of Vuse e-cigarettes. Vuse e-cigarettes were chosen as the comparison group since, at the time of data collection, Vuse was the second leading e-cigarette brand in the U.S. market.[4] The series of questions began with the instruction “Now we’d like you to compare JUUL to Vuse, another brand of e-cigarettes (pictured below).” The respondents were then asked to select the brand (JUUL or Vuse) they thought to be “more fun to use,” “better tasting,” “liked more by friends,” “cooler,” “better at helping someone to quit smoking,” “less harmful to the health of the user,” “less harmful to people next to the user,” and “less addictive.” Respondents also had the option of indicating that the two brands were “about the same.” Responses were recoded to indicate whether respondents preferred JUUL (1) vs. preferred Vuse or thought both brands were the same (0) across various attributes.

#### JUUL e-cigarette use

JUUL e-cigarette use was measured by asking “Have you ever used a JUUL, even just one time?” with the following response options: “I have heard of JUUL but I have never used it before,” “I have used JUUL before but more than 30 days ago,” and “I have used JUUL in the last 30 days.” Two variables for JUUL use were created based on this question: (1) ever use vs. never use and (2) past-30-day (current) use vs. all others.

#### Covariates

Respondents’ socio-demographic characteristics, cigarette smoking history, and nicotine dependence levels were used as covariates for this study. Socio-demographic characteristics included age (ages 18–30 and ≥31), gender (Male and Female), race/ethnicity (Non-Hispanic White, Non-Hispanic Black, Non-Hispanic Other, and Hispanic), education (≤High school/vocational, Some college, and ≥College graduate), employment (Employed and Other), marital status (Married, Single, and Other), and annual household income (<US$50,000, and ≥US$50,000). The categories for cigarette smoking status were current non-daily smokers who smoked less than 30 days in the past month and current daily smokers who smoked every day in the past 30 days. Nicotine dependence was measured based on the Fagerström Test for Nicotine Dependence and was categorized as “low” if the respondents were scored between 0 and 4, “medium” if scored between 5–6, and “high” if scored between 7 and 10 [27].

### Statistical analyses

Data were analyzed using Stata 14.0 (StataCorp: College Station, TX). First, descriptive statistics were calculated to describe respondents’ socio-demographic characteristics, cigarette smoking history, nicotine dependence, sources of awareness about JUUL e-cigarettes, and perceptions about JUUL products as compared to Vuse. Second, multivariable logistic regression models were applied to examine the associations between receiving JUUL information through specific sources of awareness and perceiving JUUL as being more favorable than Vuse, adjusting for covariates. For these models, we examined five general categories of sources of awareness in addition to ten individual sources. Third, separate multivariable logistic regression models were used to examine the relationships between each type of JUUL-related perceptions and JUUL use behaviors (ever use and current use), adjusting for covariates. Missing values for all variables were reported in Table 1. Less than 5% of the overall sample (n = 17) had missing values in one or more measures. The observations with missing values, therefore, were deleted from the individual models by listwise deletion [28].

**Table 1:** Characteristics of study samples who were aware of JUUL among adult smokers (N = 341).

			n (%)
**Age**			
	18–30		145 (42.5%)
	≥31		196 (57.5%)
**Gender**			
	Male		195 (57.2%)
	Female		146 (42.8%)
**Race/ethnicity**			
	Non-Hispanic White		269 (78.9%)
	Non-Hispanic Black		19 (5.6%)
	Non-Hispanic Other		26 (7.6%)
	Hispanic		27 (7.9%)
**Education**			
	≤High school/vocational		77 (22.6%)
	Some college		85 (24.9%)
	≥College graduate		178 (52.3%)
	Missing		1 (0.3%)
**Employment**			
	Employed		264 (77.4%)
	Other		77 (22.6%)
**Marital Status**			
	Married		231 (67.7%)
	Single		53 (15.5%)
	Other		54 (15.8%)
	Missing		3 (0.9%)
**Annual Household Income**			
	<$50K		91 (26.7%)
	≥$50K		249 (73.0%)
	Missing		1 (0.3%)
**Cigarette Smoking Status**			
	Current non-daily smoker		139 (40.8%)
	Current daily smoker		202 (59.2%)
**Nicotine Dependence**			
	Low (score 0–4)		24 (7.1%)
	Medium (score 5–6)		113 (33.1%)
	High (score 7–10)		204 (59.8%)
**JUUL Use Status**			
	Never use		195 (57.2%)
	Ever, but not in the past 30 days		65 (19.0%)
	Past-30-day use		79 (23.2%)
	Missing		2 (0.6%)
**Sources of Awareness about JUUL E-cigarettes**			
	**General advertising**		226 (66.3%)
		Print media ads (magazine/newspaper)	85 (24.9%)
		Internet ad (online banner/social media)	180 (52.8%)
		Direct mail/email	51 (15.0%)
	**JUUL brand advertising**		107 (31.4%)
		JUUL sponsored events	51 (15.0%)
		JUUL outdoor ads	38 (11.1%)
		JUUL website or social media account	57 (16.7%)
	**Social circle**		103 (30.2%)
		Word-of-mouth from friends and family	67 (19.7%)
		Friends and family’s social media account	44 (12.9%)
	**News stories on TV/radio/online**		52 (15.3%)
	**Physical Store**		26 (7.6%)

1. Ten individual sources of awareness about JUUL e-cigarettes were grouped into five categories.

## Results

### Respondent characteristics, sources of awareness, and perceptions of using JUUL

Table 1 shows that 42.5% of the respondents were between the ages of 18 and 30. More than half (57.2%) of the respondents were male. Most (78.9%) of the respondents were non-Hispanic White. In terms of JUUL use history, 42.8% of the respondents had used JUUL before, 19.0% reported having used JUUL more than a month ago, and 23.2% reported currently using JUUL. Table 2 shows sources of awareness of JUUL e-cigarettes (by five general categories and ten individual sources) as well as the comparative perceptions between JUUL and Vuse e-cigarettes. Internet advertisements (52.8%) were the most popular source of awareness related to JUUL followed by print media advertisements (24.9%). The most commonly endorsed comparative perceptions about the two e-cigarette brands were that JUUL tasted better (49.1%) and JUUL was cooler (48.6%).

**Table 2:** Perceptions about JUUL as compared to Vuse e-cigarettes (n = 341).

	Comparative Perceptions of Using JUUL vs. Vuse							
	More fun to use	Tastier	My friends would like more	Cooler	Better help quitting smoking	Less harmful to users	Less harmful to bystanders	Less addictive
	n (%)	n (%)	n (%)	n (%)	n (%)	n (%)	n (%)	n (%)
JUUL	115 (34.5%)	163 (49.1%)	120 (36.1%)	158 (48.6%)	145 (44.2%)	116 (35.4%)	106 (32.5%)	108 (32.9%)
About the same	109 (32.8%)	96 (28.9%)	122 (36.8%)	108 (33.2%)	118 (36.0%)	137 (41.8%)	141 (43.3%)	148 (45.1%)
Vuse	108 (32.5%)	73 (22.0%)	90 (27.1%)	59 (18.2%)	65 (19.8%)	75 (22.8%)	79 (24.2%)	72 (22.0%)

1. Each perception variable has missing data smaller than 5% of the overall sample size.

### The relationships between sources of awareness and perceptions of using JUUL

As Table 3 shows, several sources of awareness were significantly associated with favorable perceptions about using JUUL as compared to using Vuse, controlling for covariates. Most notably, learning about JUUL information through general advertising was associated with perceiving JUUL as tastier (AOR = 3.45, 95% CI = 1.96, 6.07), better to help quitting smoking (AOR = 2.01 95% CI = 1.13, 3.55), less harmful to users (AOR = 2.39, 95% CI = 1.28, 4.45), and less harmful to bystanders (AOR = 2.54, 95% CI = 1.33, 4.83) than Vuse. In regard to the results related to specific sources of awareness, receiving JUUL information through internet ads were associated with perceiving JUUL as more fun to use (AOR = 2.04, 95% CI = 1.21, 3.42), tastier (AOR = 1.96, 95% CI = 1.19, 3.22) and less harmful to bystanders (AOR = 1.74, 95% CI = 1.01, 2.99) as compared to Vuse. Initially receiving JUUL information through print media ads was associated with perceiving JUUL as more helpful in quitting smoking (AOR = 2.03, 95% CI = 1.33, 3.65) and less harmful to bystanders (AOR = 2.52, 95% CI = 1.41, 4.55) than Vuse. The respondents who initially received information through word-of-mouth were less likely to perceive JUUL as less harmful to users (AOR = 0.49, 95% CI = 0.24, 0.96) as compared to Vuse. Additionally, those who initially received JUUL information through news stories were less likely to perceive JUUL as tastier (AOR = 0.31, 95% CI = 0.15, 0.65) or better liked by their friends (AOR = 0.33, 95% CI = 0.14, 0.75) relative to Vuse.

**Table 3:** Comparative perceptions of using JUUL vs. Vuse and sources of awareness about JUUL among adult smokers (N = 341).

		Comparative Perceptions of Using JUUL vs. Vuse								
		More fun to use	Tastier		My friends would like more	Cooler	Better help quitting smoking	Less harmful to users	Less harmful to bystanders	Less addictive
		AOR (95% CI)	AOR (95% CI)		AOR (95% CI)	AOR (95% CI)	AOR (95% CI)	AOR (95% CI)	AOR (95% CI)	AOR (95% CI)
**General advertising**		1.30 (0.74, 2.31)		**3.45 (1.96, 6.07)**	0.96 (0.54, 1.70)	1.41 (0.80, 2.48)	**2.01 (1.13, 3.55)**	**2.39 (1.28, 4.45)**	**2.54 (1.33, 4.83)**	1.84 (0.96, 3.53)
	Print media ads	0.73 (0.41, 1.33)		1.62 (0.91, 2.87)	0.61 (0.34, 1.09)	0.86 (0.48, 1.55)	**2.03 (1.33, 3.65)**	1.05 (0.58, 1.87)	**2.52 (1.41, 4.55)**	0.66 (0.36, 1.22)
	Internet ads	**2.04 (1.21, 3.42)**		**1.96 (1.19, 3.22)**	0.86 (0.51, 1.43)	1.33 (0.80, 2.23)	1.62 (0.98, 2.68)	1.51 (0.89, 2.57)	**1.74 (1.01, 2.99)**	1.67 (0.96, 2.90)
	Direct mail/email	0.47 (0.23, 0.99)		1.59 (0.80, 3.16)	0.78 (0.39, 1.56)	1.56 (0.70, 3.17)	0.87 (0.45, 1.72)	0.74 (0.38, 1.49)	1.19 (0.59, 2.40)	1.29 (0.64, 2.57)
**JUUL brand advertising**		0.93 (0.53, 1.64)		0.80 (0.45, 1.41)	1.12 (0.63, 1.95)	1.79 (0.92, 3.09)	1.60 (0.92, 4.15)	1.84 (1.05, 3.24)	1.28 (0.72, 2.29)	1.56 (0.88, 2.80)
	JUUL sponsored events	0.43 (0.26, 1.10)		0.62 (0.31, 1.23)	1.08 (0.55, 2.12)	1.48 (0.72, 3.01)	0.88 (0.44, 1.75)	0.89 (0.45, 1.76)	1.47 (0.74, 2.92)	1.10 (0.55, 2.19)
	JUUL outdoor ads	1.00 (0.47, 2.12)		1.37 (0.64, 2.95)	**0.41 (0.17, 0.95)**	1.16 (0.52, 2.56)	2.10 (0.95, 4.64)	2.08 (0.97, 4.47)	1.13 (0.52, 2.45)	2.03 (0.93, 4.44)
	JUUL website/social media	1.07 (0.56, 2.07)		0.76 (0.40, 1.47)	1.21 (0.63, 2.32)	1.70 (0.84, 3.41)	0.86 (0.45, 1.66)	1.23 (0.65, 2.37)	1.19 (0.61, 2.31)	1.45 (0.74, 2.81)
**Social circle**		0.88 (0.52, 1.48)		1.40 (0.83, 2.33)	1.58 (0.95, 2.64)	1.58 (0.95, 2.64)	1.33 (0.79, 2.24)	**0.54 (0.31, 0.96)**	1.21 (0.70, 2.09)	0.64 (0.36, 1.16)
	Word-of-mouth	0.66 (0.35, 1.24)		1.40 (0.77, 2.52)	1.56 (0.86, 2.82)	1.29 (0.70, 2.36)	1.04 (0.57, 1.90)	**0.49 (0.24, 0.96)**	0.89 (0.46, 1.70)	0.53 (0.26, 1.08)
	Friends and family’s social media	1.24 (0.62, 2.51)		1.09 (0.55, 2.19)	1.54 (0.76, 3.05)	0.60 (0.25, 1.05)	1.47 (0.73, 2.96)	0.83 (0.39, 1.73)	**2.10 (1.03, 4.26)**	1.17 (0.55, 2.47)
**News stories on TV/radio/online**		0.74 (0.37, 1.49)		**0.31 (0.15, 0.65)**	**0.33 (0.14, 0.75)**	0.51 (0.25, 1.03)	0.73 (0.36, 1.49)	0.50 (0.23, 1.11)	0.57 (0.25, 1.29)	0.43 (0.18, 1.03)
**Physical Store**		1.15 (0.47, 2.78)		0.87 (0.36, 2.12)	0.60 (0.22, 1.65)	0.40 (0.15, 1.07)	0.42 (0.15, 1.15)	**0.15 (0.03, 0.69)**	0.41 (0.13, 1.31)	0.31 (0.08, 1.13)

1. The regression model for each source of awareness controlled for covariates.

2. The reference group of those models was having no exposure to the particular source of awareness

3. The models examined the exposure to five general categories of sources and ten individual sources.

### The relationships between perceptions of using JUUL and JUUL use behaviors

Table 4 presents the associations between the perceptions of using JUUL and the behavior of using JUUL, controlling for covariates. Results showed that perceiving JUUL as being tastier (AOR = 2.07, 95% CI = 1.23, 3.49), more helpful for quitting smoking (AOR = 2.07, 95% CI = 1.22, 3.53), and cooler (AOR = 2.07, 95% CI = 1.21, 3.56) than Vuse was associated with ever using JUUL. Only perceiving JUUL as being tastier (AOR = 1.98, 95% CI = 1.10, 3.59) than Vuse was associated with current use of JUUL.

**Table 4:** Comparative perceptions of using JUUL vs. Vuse and JUUL use behavior among adult cigarette smokers (N = 341).

		JUUL Use Behavior			
		Ever Use Vs. Never Use		Current Use vs. All Other	
Comparative Perceptions of Using JUUL vs. Vuse		N (%)	AOR (95% CI)	N (%)	AOR (95% CI)
	More fun to use	49 (43.0%)	0.96 (0.57, 1.62)	27 (23.7%)	0.95 (0.53, 1.71)
		93 (65.5%)	Reference	51 (23.1%)	Reference
	Tastier	90 (55.2%)	**2.07 (1.23, 3.49)**	50 (30.7%)	**1.98 (1.10, 3.59)**
		51 (30.3%)	Reference	26 (15.5%)	Reference
	My friends would like more	62 (51.7%)	1.67 (0.98, 2.84)	34 (28.3%)	1.35 (0.76, 2.42)
		77 (36.5%)	Reference	41 (19.4%)	Reference
	Cooler	85 (45.9%)	**2.07 (1.21, 3.56)**	45 (28.7%)	1.27 (0.70, 2.30)
		53 (31.7%)	Reference	30 (18.0%)	Reference
	Better help quitting smoking	82 (56.9%)	**2.07 (1.22, 3.53)**	45 (31.3%)	1.49 (0.83, 2.67)
		57 (31.2%)	Reference	30 (16.4%)	Reference
	Less harmful to users	62 (53.5%)	1.24 (0.72, 2.13)	33 (28.5%)	1.09 (0.60, 1.98)
		76 (36.0%)	Reference	41 (19.4%)	Reference
	Less harmful to bystanders	58 (55.2%)	1.29 (0.74, 2.24)	36 (34.3%)	1.58 (0.87, 2.87)
		81 (36.8%)	Reference	39 (17.7%)	Reference
	Less addictive	58 (53.7%)	1.04 (0.59, 1.83)	32 (29.6%)	1.02 (0.55, 1.88)
		80 (36.5%)	Reference	42 (19.2%)	Reference

1. The regression model for each perception of JUUL products controlled for covariates.

2. Each perception was included in separate regression models.

## Discussion

This study contributes to the limited knowledge about adult cigarette smokers’ perceptions of JUUL e-cigarettes, the sources of awareness that potentially drive these perceptions, and the associations between such perceptions and use of JUUL. Our results indicated that even for adult cigarette smokers, the perceived benefits of fun and sensory experience from using JUUL may outweigh the perceived benefits of smoking cessation, at least when comparing JUUL against Vuse (a competitor brand). Those positive sensory and recreational perceptions of using JUUL, rather than positive smoking cessation benefits, are more likely to sustain JUUL use behavior. Additionally, adult smokers first learn about most of those positive benefits of using JUUL through internet ads (including online banners and social media), a similar method for young, naïve tobacco users to learn about JUUL products as indicated by previous studies [21,29].

This study found that although JUUL and Vuse both produce popular pod-shaped and nicotine salt-based e-cigarettes that come in similar flavors, adult cigarette smokers perceived JUUL more positively as compared to Vuse in many domains. The two most endorsed items (JUUL is tastier and cooler than Vuse) signified that adult smokers value the sensory and social experience from using JUUL more so than Vuse. This finding suggests that although JUUL Labs claimed that the thrust of their marketing message is that JUUL provides adult smokers with an alternative to smoking cigarettes [7], adult smokers’ JUUL use appears to be centered more so on the product’s “fun” and “cool” aspects.

Our study also found that learning about JUUL e-cigarettes through general advertising, particularly internet ads including online banners and social media, was associated with having the most types of positive perceptions about using JUUL (e.g., enhanced sensory experience and harm reduction) among adult smokers. This finding signified the potential influence of initial exposure to JUUL information through internet ads on shaping smokers’ beliefs about JUUL products. These internet ads for JUUL e-cigarettes may be created and disseminated by JUUL Labs, social media influencers or brand ambassadors sponsored by JUUL Labs [21], and/or e-cigarette manufacturers and vendors that produce, market, and sell JUUL-compatible products such as flavored pods [30]. Therefore, although JUUL removed their official Facebook and Instagram accounts in November 2018 in response to FDA’s enforcement actions [26], our findings indicate that this change may not greatly reduce the influence of JUUL-related advertisements and promotions through general advertising, particularly through the internet and social media platforms. Future studies are needed to distinguish JUUL e-cigarette ads created by social media influencers from those generated by JUUL’s official outlets in order to understand the underlying differences in the messages disseminated by these two types of outlets. Additionally, the U.S. Federal Trade Commission (FTC) could strengthen its enforcement of regulations requiring disclosure of sponsored commercial activities on the internet (including social media influencers) [31] and, in doing so, would help consumers (including smokers and non-smokers) make informed decisions about using JUUL products.

Our study finding also indicated that favorable perceptions of JUUL’s sensory appeals, rather than perceptions about harm reduction or smoking cessation as compared to Vuse, were associated with current JUUL use among adult smokers. The favorable taste of JUUL products may be attributable to the presence of nicotine salt, which enhances the palatability of e-cigarette aerosol as well as the attractiveness of its flavors [32]. Evidence has suggested that e-cigarette flavors are associated with increased e-cigarette use frequency and quantity among adults and cigarette smokers [33,34]. The facts that JUUL e-cigarettes come with popular pre-filled flavors (e.g., mint and mango) [32] and JUUL-compatible products offer a large number of flavor options [30] may increase users’ positive sensory perceptions from using JUUL. Furthermore, our study found that perceiving JUUL as more helpful as compared to Vuse for quitting smoking was associated with JUUL experimentation rather than the current use. A possible explanation for this finding may be that JUUL is viewed as better for helping to quit among experimenters (vs. never users) but similarly helpful for quitting among current users (vs. non-current users) as compared to Vuse. Alternatively, other motivating factors (such as flavors) may have played a more important role in sustaining JUUL use among our respondents than Vuse. Since the FDA continues to crackdown on the availability of e-cigarette flavors [35] and a growing number of localities restrict the sale of flavored e-cigarettes [36], more research is needed to further inform the regulation by examining the role of JUUL’s flavors in encouraging smokers to transition to and continue to use JUUL products. Nevertheless, since only 23.2% (n = 79) of our respondents were currently using JUUL, this study may be underpowered to detect the associations between other JUUL related perceptions and current JUUL use.

This study should be viewed with the following limitations. First, the study relied on an online sample that may be subject to selection bias; for example, the respondents who participated in online studies may hold certain characteristics that put them at different risks of using JUUL compared to the general population. However, the prevalence of ever and current JUUL use among adult smokers in our study is comparable to that of a nationally representative study [5] and an online study using a large U.S. sample of adult combustible users [6], signifying that our data may resemble the pattern of using JUUL among adult smokers in the U.S. Nevertheless, our findings need to be further confirmed with studies using large samples. Second, this study’s data collection took place before JUUL took a series of actions on their marketing strategies in November 2018, such as removing some of its official social media accounts and using models of older ages [26]. Therefore, the prevalence of JUUL-related information in various media channels as well as the content of JUUL marketing materials may have changed over time. Third, although we included pictures of Vuse e-cigarettes in the survey, we did not know whether the respondents in our study had heard of Vuse e-cigarettes prior to the survey. Respondents were allowed to skip the questions if they did not know what Vuse was or did not wish to respond to the questions related to Vuse. Additionally, since our sample was restricted to the respondents who had heard of JUUL e-cigarettes before, it was likely that they had also heard of Vuse e-cigarettes, since Vuse was the second market leader at the time of survey collection. Lastly, our survey only captured the comparative perceptions of JUUL vs. Vuse e-cigarettes and did not assess participants’ absolute perceptions of JUUL e-cigarettes. Therefore, our findings do not indicate whether absolute perceptions of JUUL e-cigarettes are associated with JUUL use behavior among adult smokers. Future research is recommended to also assess the absolute perceptions of e-cigarette brands to enhance the understanding of adult cigarette smokers’ overall and comparative perceptions towards various e-cigarette products and brands.

Our study has multiple research and policy implications for understanding and leveraging the influence of JUUL’s marketing strategies in enhancing public health benefits among adult cigarette smokers. Although no conclusive evidence has shown e-cigarettes, including JUUL, successfully help cigarette smokers reduce or quit cigarette smoking, only using e-cigarettes or JUUL products was proven to be less harmful than e-cigarette and cigarette dual use or smoking cigarettes [10,37]. Pending further scientific proof, JUUL e-cigarettes may hold the potential of helping smokers transition off of cigarettes and reducing and quitting cigarette smoking [8,9]. Given the findings from our study that adult smokers may be more likely to focus on the sensory and recreational experience of using JUUL rather than JUUL’s smoking cessation benefits, marketing regulations aimed at minimizing JUUL’s appeal among young, naïve tobacco users may need to take into account the unintended consequences of those adjustments among adult cigarette smokers. Specifically, regulating social media accounts may reduce the influence of JUUL’s marketing strategies among young, naïve tobacco users, it may also diminish the positive perceptions about JUUL products among adult cigarette smokers.

Additionally, more evidence-based strategies are needed in place to enhance the appeal and influence of smoking cessation claims among adult smokers. The same tactics (e.g., social media influencers) that the e-cigarette and tobacco industry has adopted to attract young, naïve tobacco users may be adjusted and adapted to encourage adult smokers to transition to exclusively using e-cigarette products. More research is also needed to assess whether JUUL’s sensory (e.g., flavors) and social (e.g., fun and cool to use) appeals are more likely to encourage adult smokers to initiate and sustain JUUL use over time as compared to its smoking cessation appeals, and whether these varying perceptions are associated with short-term and long-term cigarette smoking cessation. Finally, before conclusive evidence shows JUUL’s effectiveness in helping smokers quit smoking, messages about the high nicotine addiction potential from using JUUL [10] and recommendations for using FDA approved smoking cessation methods are greatly needed to help adult smokers make informed decisions on smoking reduction and cessation.

## Conclusions

This study concludes that adult smokers highly value the sensory and recreational appeals of JUUL use and that learning about JUUL through internet ads is associated with having many positive perceptions of using JUUL than Vuse. These sensory and recreational and recreational perceptions of using JUUL, in turn, are associated with JUUL ever and current use. Regulating JUUL’s internet marketing to minimize its appeals among tobacco-naïve young people may inadvertently diminish the positive perceptions and use of JUUL e-cigarettes among adult smokers.
